# Comparison of Clinical, Pathological, and Procedural Characteristics of Adult and Pediatric Acute Appendicitis before and during the COVID-19 Pandemic

**DOI:** 10.3390/children11060641

**Published:** 2024-05-25

**Authors:** Goran Augustin, Jurica Žedelj, Karmen Jeričević, Nora Knez

**Affiliations:** 1 Department of Surgery, University Hospital Centre Zagreb, 10000 Zagreb, Croatia; 2 School of Medicine, University of Zagreb, 10000 Zagreb, Croatia; 3 Varaždin General Hospital, Ul. Ivana Meštrovića 1, 42000 Varaždin, Croatia; 4 Institute of Emergency Medicine of the City of Zagreb, Ul. Vjekoslava Heinzela 88, 10000 Zagreb, Croatia

**Keywords:** acute abdomen, acute appendicitis, appendectomy, COVID-19 pandemic

## Abstract

Background: This study aims to analyze the impact of the COVID-19 pandemic on the clinical, pathological, and surgical characteristics of acute appendicitis (AA) at the University Hospital Centre (UHC) Zagreb. Methods: This retrospective study analyzed demographic, clinical, and surgical data from consecutive AA patients. Data were collected from an electronic database for two periods: 1 January to 31 December 2019 (pre-COVID-19), and 11 March 2020, to 11 March 2021 (COVID-19 pandemic). Results: During the two study periods, 855 appendectomies were performed, 427 in the pre-pandemic, and 428 during the pandemic. Demographic data were comparable between groups. There was statistically no significant difference in the type of appendectomy (*p* = 0.33) and the median hospital length of stay (3; (2–5) days, *p* = 0.08). There was an increase in the conversion rate during the pandemic period (4.2% vs. 7.7%, *p* = 0.03). The negative appendectomy rate and the incidence of perforated AA did not differ significantly (*p* = 0.34 for both). Conclusions: We did not observe a significant increase in the rate of AA complications during the COVID-19 pandemic at the UHC Zagreb. This may be attributed to two factors: (1) AA was diagnosed and treated as an emergency, which remained available during the pandemic, and (2) diagnostic and therapeutic protocols remained unaltered. We recommend a laparoscopic approach even during the COVID-19 pandemic.

## 1. Introduction

Acute appendicitis (AA) constitutes a prevalent emergency within abdominal surgery, boasting an estimated lifetime risk of 6.7% to 8.6% [1]. In a recent meta-analysis, the occurrence rates were calculated to be 100, 105, and 151 per 100,000 person-years in North America, Eastern Europe, and Western Europe, respectively [2]. The frequency of acute pediatric appendicitis in the 21st century varies between 100 and 151 cases per 100,000 person-years, with variations observed based on geographical location [3]. The highest risk group for appendectomy comprises adolescent girls aged 12 to 16 years [4]. A widely used classification divides AA into uncomplicated and complicated; complicated refers to gangrenous or perforated AA or pelvic/abdominal abscess [5]. Appendectomy remains the gold standard for treating AA, as opposed to non-operative management (NOM) with antibiotics, making it a frequent surgical procedure [6,7].

COVID-19 (COronaVIrus Disease-19) is an infectious disease caused by the coronavirus SARS-CoV-2, initially recognized in December 2019 in China [8]. Rapid global dissemination prompted the World Health Organization (WHO) to proclaim a pandemic on 11 March 2020 [9]. The first case in Croatia was confirmed on 25 February 2020.

Throughout the pandemic, there was a notable decline in individuals seeking medical care for non-COVID-19-related emergencies, including conditions like acute coronary syndrome, stroke, and acute abdomen. From March to April 2020, hospitals worldwide experienced a 42% drop in emergency department visits, with a significant decrease in visits for abdominal pain [10]. The pandemic mandated changes in hospital protocols, including waiting for COVID-19 PCR results, which determined further organizational and management changes. Multiple studies have shown that this affected the management of AA.

A systematic review and meta-analysis by Köhler et al. showed a decrease of AA cases by 20.9% in adults but an increase of 13.4% in children. There was also an increased rate of complicated AA during the pandemic [11]. In the pediatric population, a higher incidence of complicated AA was observed. Moreover, a higher proportion of children was treated by NOM [12]. On the other hand, a retrospective monocenter observational study from UHC Rijeka, Rijeka, Croatia, showed no significant difference in the duration of symptoms and the incidence of complicated AA between the pre-COVID and COVID period [13].

Despite the widespread impact of the COVID-19 pandemic, the research conducted by Bosak Veršić et al. stands out as the sole study investigating the implications of AA during this period in Croatia, based on our current knowledge. Due to the scarcity of research, further investigations are needed to comprehensively assess the clinical presentation, management approaches, and outcomes of AA during the COVID-19 pandemic in Croatia.

## 2. Materials and Methods

The data were retrospectively collected from the electronic database for consecutive patients admitted for appendectomy at the Department of Surgery at University Hospital Centre Zagreb (UHC) Zagreb, Zagreb, Croatia (Supplementary Materials). Inclusion criteria consisted of patients diagnosed with acute appendicitis (AA), while exclusion criteria included all other groups with abdominal complaints and diagnoses. Patients were divided into two comparable groups: the pre-COVID-19 group, defined as patients treated before the COVID-19 pandemic (from 1 January 2019, to 31 December 2019), and the COVID-19 group, defined as patients treated during the pandemic (from 11 March 2020, to 11 March 2021). The selected period corresponds to the timeframe when the strictest restrictions were imposed, ensuring that our analysis captures the potential impact of these measures on AA outcomes. The patients were stratified and compared according to age groups. Pediatric patients were younger than 16. The age cutoff of 16 years was chosen to differentiate between pediatric and adult patients. This criterion reflects the longstanding practice at the UHC Zagreb, where patients with AA younger than 16 are admitted to the pediatric surgery division, while those older than 16 are to two abdominal surgery divisions. This categorization has been traditionally followed for the past 50 years. At UHC Zagreb, adult AA is managed by abdominal surgeons and pediatric AA by pediatric surgeons or abdominal surgeons, depending on the 24-h emergency shifts. Appendectomy is indicated in patients with persistent right lower quadrant abdominal pain, clinical signs (e.g., rebound tenderness, McBurney’s point tenderness), and suggestive laboratory findings (elevated white blood cell count, C-reactive protein levels), along with imaging consistent with acute appendicitis. Discharge criteria post-appendectomy include resolution of pain and tenderness, normal bowel function, stable vital signs, ability to tolerate oral intake and pass flatus or stool, adequate pain control, and understanding of postoperative instructions. Pain management post-appendectomy involves multimodal analgesia, including NSAIDs, paracetamol, and opioids, as needed. To prevent complications, oral intake progresses from clear liquids to a regular diet, avoiding heavy or greasy foods initially. Follow-up within 1–2 weeks assesses wound healing and monitors for complications. Complications were categorized using the Clavien-Dindo classification system.

Electronic medical records were reviewed for demographic (age, sex), clinical (duration of hospitalization), pathological (gangrenous AA, perforated AA, complications), and surgical (type of appendectomy, negative appendectomy, conversion, reoperation) data. All appendectomies at UHC Zagreb start as laparoscopy. The primary objective was to compare clinical, pathological, and surgical characteristics of AA before and during the COVID-19 pandemic to evaluate the impact of the pandemic on AA complications incidence. The secondary objective was to compare those characteristics separately for the pediatric and adult populations.

All data were collected into the EXCEL database (v.2022, Microsoft, Washington, DC, USA). The statistical analysis was performed using SPSS version 29.0.0.0 (SPSS, Inc., Chicago, IL, USA). The normality of distribution for quantitative variables was tested using the Kolmogorov-Smirnov test. Continuous data are presented as median with interquartile range (IQR) and compared between groups using the Mann-Whitney U test. Categorical data are presented as absolute numbers with percentages and compared between groups using the χ^2^ (chi-square) test or Fisher’s exact test when the number of events was <5. A *p*-value of <0.05 was considered statistically significant.

## 3. Results

### 3.1. All Patients

During the two study periods, 855 appendectomies were performed: 427 in the pre-pandemic period and 428 during the pandemic. The median age of the entire cohort was 27 (16–42) years, and 450 (52.6%) patients were males (Table 1). Demographic characteristics did not significantly differ between the groups.

During the pandemic period, a significantly higher conversion rate was observed (4.2% vs. 7.7%, *p* = 0.03). The difference in the appendectomy approach (open or laparoscopic), length of hospitalization, reoperation rates, negative appendectomy rate, and incidence of perforated AA or other complications was not statistically significant between the two groups. There were no reoperations during primary hospitalizations.

The types of complications and Clavien-Dindo classifications are shown in Table 2 and Table 3, respectively.

### 3.2. Adult Patients

The study included 630 adult patients who underwent appendectomy, 313 patients in the pre-pandemic group, and 317 in the pandemic group (Table 4). Demographic data were comparable between the groups. The pandemic group exhibited a significant increase in the conversion rate (4.8% pre-pandemic vs. 8.8% during the pandemic, *p* = 0.04). There was no statistically significant difference in the appendectomy approach, length of hospitalization, reoperation, negative appendectomy rates, and the incidence of gangrenous, perforated AA, or other complications between the groups.

### 3.3. Pediatric Patients

The study included 225 pediatric patients with AA, 114 during the pre-pandemic period and 111 during the pandemic (Table 5). Demographic characteristics did not differ significantly between the groups. There was no significant difference in the choice of appendectomy approach, median length of hospital stay, conversion and reoperation rates, negative appendectomy rates, perforated AA rates, and other complications between the groups.

## 4. Discussion

The study did not identify any notable variations in the clinical, pathological, and surgical characteristics among AA patients before and during the COVID-19 pandemic. Numerous factors are likely involved in the development of complicated AA, including the presence of appendicolith, delayed surgery following unsuccessful non-operative management, and prolonged symptom duration [14,15]. Numerous studies have documented a rise in the incidence of perforated AA during the COVID-19 pandemic. We did not observe an increased rate of complicated AA. Our study’s findings diverge from those of Kariya et al.’s meta-analysis, which emphasized an elevated incidence of complicated AA during the COVID-19 pandemic [16]. Wang et al., for example, reported that 31% of their cohort of 80 AA patients presented with perforated AA during the pandemic [17]. Kupietzky et al. reported a significant increase in the number of patients with complicated and perforated AA during the first year of the COVID-19 pandemic. However, they found that clinical outcomes remained unaffected [18]. We did not observe an increased rate of complicated pediatric AA during the COVID-19 pandemic, as discussed in the meta-analysis conducted by Pogorelić et al. [12]. Our outcomes are consistent with those of a similar study from UHC Rijeka, Croatia, indicating no discernible difference in the rate of AA complications before and during the pandemic [13].

Conversion rates are within the published series [19,20]. It should be considered that all appendectomies at UHC Zagreb start as laparoscopy, increasing the likelihood of conversion. The predominant reasons for conversion were complicated intraoperative findings and the inability to find the appendicular base due to inflammatory changes. Rarely was conversion needed due to equipment issues. Despite stable rates of complicated AA, there was a notable increase in conversion rates during the pandemic (4.2% vs. 7.7%, *p* = 0.03). After stratifying patients into pediatric and adult populations, a statistically significant increase in conversion rates was observed for adult patients (4.8% to 8.8%, *p* = 0.04).

This discrepancy can be attributed to surgeons being less likely to proceed with technically more challenging laparoscopic surgeries, thus prolonging the duration of the procedure and increasing the chances of complications. Additionally, logistical challenges and resource constraints within healthcare systems during the pandemic may have influenced surgical decision-making, increasing conversion rates.

Our criteria for a negative appendectomy are based on the histopathological findings. This is the standard practice at UHC Zagreb. The results from Bosak Veršić et al. demonstrated a negative appendectomy rate of 11.24% overall, with rates of 13.75% and 8.02% observed in the pre-COVID-19 and COVID-19 eras, respectively. In comparison, our findings show that the negative appendectomy rates were 4.9%, 5.6%, and 4.2% in the respective periods (*p* = 0.34). A study by Jukić et al. also shows a higher overall negative appendectomy rate for pediatric patients during a ten-year period [21]. The reported negative appendectomy rate varies considerably in the literature, mainly due to poorly defined inclusion criteria [22].

The reasons for revisional surgery include intra-abdominal collections, bleeding, abdominal wall dehiscence, and bowel obstruction [23]. In our center, the revision rates were 1.1% overall, 0.6% in the pre-COVID-19 group, and 1.6% in the COVID-19 group (*p* = 0.45).

The onset of the SARS-CoV-2 pandemic has led to substantial global changes in routine healthcare and daily life [24]. Delayed presentation of AA leads to a more complicated AA, one of the risk factors for conversion from a laparoscopic to an open approach [6]. The increased conversion rates during the pandemic could be due to factors unrelated to the severity of AA, such as the changes in surgical teams performing the procedure.

AA is a common surgical condition with relatively stable incidence in Western countries, making it a good indicator of the COVID-19 pandemic influence [25]. Our study is the most comprehensive analysis of AA outcomes during the COVID-19 pandemic in Croatia, and the results can be discussed in several ways. The healthcare system in Croatia was not as burdened by the COVID-19 pandemic as in some other countries, which may have affected the continuity of healthcare for patients with AA. The results indicate that AA was still recognized as an emergency requiring prompt medical intervention, and emergency medical care and surgical protocols remained unchanged during the COVID-19 pandemic.

During the pandemic, most medical centers worldwide postponed or canceled regular elective procedures, and the same trend was applied in our institution. Surgical procedures were limited to emergency cases and patients with malignant diseases to reduce unnecessary burdens on the healthcare system and allow enough beds for COVID-19-positive patients. Additionally, a separate operating room was set up in the premises of the unified emergency hospital admission department for COVID-19-positive patients due to the risk of virus transmission through the medical gas pipeline system and contamination of the operating rooms. Until 31 October 2021, all COVID-19-positive patients underwent surgery at another medical center reorganized for COVID-19-positive patients. From 1 November 2021 onwards, surgeries started at our facility, but in the specialized COVID ward with McBurney incision, involving a total of 4 COVID-19-positive patients. In the context of AA, there were no substantial changes in the diagnostic protocol, and patients were admitted and diagnosed through the centralized emergency hospital admission department. Due to the concerns regarding the safety of laparoscopic surgery on COVID-19-positive patients, only open-approach appendectomies were performed on COVID-19 patients at the beginning of the pandemic. Given recent evidence affirming the safety of laparoscopy, laparoscopic appendectomy became the preferred treatment for all patients [26].

The rate of NOM increased worldwide amid the pandemic [27]. Regarding uncomplicated AA, the 1-year success rate of NOM is lower than that of surgical treatment. However, delayed appendectomy due to the failure of NOM did not increase complication rates compared to open approach [28]. At UHC Zagreb, surgical management was the sole treatment approach for AA patients during the pandemic. The NOM approach for non-complicated AA has not been widely embraced in our country. Therefore, we opted to perform appendectomy promptly for all AA patients, even amid the COVID-19 pandemic.

## 5. Conclusions

This study revealed that despite the implemented restrictions during the pandemic, there was no significant increase in the incidence of complicated AA in both adult and pediatric patients at UHC Zagreb. The absence of changes in clinical, pathological, and surgical characteristics can be attributed to the fact that AA is typically diagnosed and treated in emergency settings, which remained available during the COVID-19 pandemic. Diagnostic and therapeutic protocols for emergency conditions remained unchanged during the pandemic. The laparoscopic approach is recommended during the COVID-19 pandemic. Additionally, it can be noted that the pandemic did not strongly affect our region as it did in some other countries.

This research was presented at the 15th Congress of the Croatian Association of Digestive Surgery with International Participation and the 4th Congress of the Association of Nurses/Technicians in Digestive Surgery with International Participation held on 3–6 May 2023, in Opatija, Croatia.

## Figures and Tables

**Table 1:** All patients.

Variable	All (*n* = 855)	Pre-COVID-19 (*n* = 427)	During COVID-19 (*n* = 428)	*p*-Value
Age, median (IQR)	27 (16–42)	26 (16–41)	29 (16–44)	0.18
Pediatric patients, *n* (%)	225 (26.3)	114 (26.7)	111 (25.9)	0.80
Female, *n* (%)	405 (47.4)	216 (50.6)	189 (44.2)	0.06
Male, *n* (%)	450 (52.6)	211 (49.4)	239 (55.8)	0.06
Laparoscopic approach, *n* (%)	828 (96.8)	416 (97.4)	412 (96.3)	0.33
Hospitalization (days), median (IQR)	3 (2–5)	3 (2–5)	3 (2–5)	0.08
Conversion, *n* (%)	51 (6)	18 (4.2)	33 (7.7)	0.03
Revision, *n* (%)	7 (0.8)	2 (0.5)	5 (1.2)	0.45
Gangrenous appendicitis, *n* (%)	286 (33.5)	145 (34)	141 (32.9)	0.75
Negative appendectomy, *n* (%)	42 (4.9)	24 (5.6)	18 (4.2)	0.34
Perforated appendicitis, *n* (%)	124 (14.5)	57 (13.3)	67 (15.7)	0.34
Complications, *n* (%)	63 (7.4)	33 (7.7)	39 (7)	0.69
Complications + perforated appendicitis, *n* (%)	31 (3.6)	16 (3.7)	15 (3.5)	0.85

**Table 2:** Types of complications.

	Overall (*n* = 855)	Pre-COVID-19 Group (*n* = 427)	COVID-19 Group (n = 428)
Total complications, *n* (%)	72	33	39
Overall complication rate (%)	8.40	7.70	9.10
Wound infections, *n* (%)	7 (9.7%)	3 (9.1%)	4 (10.3%)
Abdominal wall dehiscence requiring reoperation, *n* (%)	4 (5.6%)	3 (9.1%)	1 (2.6%)
Pneumonia, *n* (%)	2 (2.8%)	2 (6.1%)	0 (0.0%)
Fever > 2 days, *n* (%)	14 (19.4%)	5 (15.2%)	9 (23.1%)
Intra-abdominal collections treated conservatively, *n* (%)	19 (26.4%)	10 (30.3%)	9 (23.1%)
Intra-abdominal collections treated with percutaneous drainage, *n* (%)	7 (9.7%)	3 (9.1%)	4 (10.3%)
Intra-abdominal collections treated surgically, *n* (%)	9 (12.5%)	4 (12.1%)	5 (12.8%)
Bowel obstruction treated conservatively, *n* (%)	4 (5.6%)	2 (6.1%)	2 (5.1%)
Bowel obstruction treated surgically, *n* (%)	5 (6.9%)	1 (3.0%)	4 (10.3%)

**Table 3:** Types of complications by Clavien-Dindo classification.

Complications	Overall (*n* = 72)	Pre-COVID-19 Group (*n* = 33)	COVID-19 Group (*n* = 39)
Clavien-Dindo II, *n* (%)	45 (62.5)	21 (63.6)	24 (61.5)
Clavien-Dindo IIIA, *n* (%)	7 (9.7%)	3 (9.1)	4 (10.3)
Clavien-Dindo IIIB, *n* (%)	17 (23.6%)	7 (21.2)	10 (25.6)
Clavien-Dindo IV, *n* (%)	2 (2.8%)	1 (3)	1 (2.6)
Clavien-Dindo V, *n* (%)	1 (1.4%)	1 (3)	-

**Table 4:** Adult patients.

Variable	All (*n* = 630)	Pre-COVID-19 (*n* = 313)	During COVID-19 (*n* = 317)	*p*-Value
Age, median (IQR)	34 (24–51)	33 (23–49)	36 (26–51)	0.08
Female, *n* (%)	319 (50.6)	168 (53.7)	151 (47.6)	0.13
Male, *n* (%)	317 (50.3)	145 (46.3)	166 (52.4)	0.13
Laparoscopic approach, *n* (%)	612 (97.1)	305 (97.4)	307 (96.8)	0.65
Hospitalization (days), median (IQR)	3 (2–5)	3 (2–5)	3 (2–5)	0.30
Conversion, *n* (%)	43 (6.8)	15 (4.8)	28 (8.8)	0.04
Revision, *n* (%)	7 (1.1)	2 (0.6)	5 (1.6)	0.45
Gangrenous appendicitis, *n* (%)	231 (36.7)	115 (36.7)	116 (36.6)	0.97
Negative appendectomy, *n* (%)	34 (5.4)	18 (5.8)	16 (5)	0.70
Perforated appendicitis, *n* (%)	99 (15.7)	46 (14.7)	53 (16.7)	0.49
Complications, *n* (%)	49 (7.8)	27 (8.6)	22 (6.9)	0.43
Complications + perforated appendicitis, *n* (%)	25 (4)	14 (4.5)	11 (3.5)	0.52

**Table 5:** Pediatric patients.

Variable	All (*n* = 225)	Pre-COVID-19 (*n* = 114)	During COVID-19 (*n* = 111)	*p*-Value
Age, median (IQR)	11 (9–14)	11 (8–14)	11 (9–14)	0.78
Female, *n* (%)	86 (38.2)	48 (42.1)	38 (34.2)	0.22
Male, *n* (%)	139 (61.8)	66 (57.9)	73 (65.8)	0.22
Laparoscopic approach, *n* (%)	216 (96.0)	111 (97.4)	105 (94.6)	0.29
Hospitalization (days), median (IQR)	4 (3–5)	4 (3–5)	4 (3–5)	0.05
Conversion, *n* (%)	8 (3.6)	3 (2.6)	5 (4.5)	0.50
Revision, *n* (%)	0	0	0	/
Gangrenous appendicitis, *n* (%)	55 (24.4)	30 (26.3)	25 (22.5)	0.51
Negative appendectomy, *n* (%)	8 (3.6)	6 (5.3)	2 (1.8)	0.28
Perforated appendicitis, *n* (%)	25 (11.1)	11 (9.6)	24 (12.6)	0.48
Complications, *n* (%)	14(6.2)	6 (5.3)	8 (7.2)	0.55
Complications + perforated appendicitis, *n* (%)	6 (2.7)	2 (1.8)	4 (3.6)	0.44

## Data Availability

The data presented in this study are available in Supplementary Materials.

## Supplementary Materials

The following supporting information can be downloaded at: https://www.mdpi.com/article/10.3390/children11060641/s1, Table S1: Appendectomy in UHC Zagreb in pre-COVID-19 and COVID-19 pandemic.
